# Electrically tunable topological phase transition in non-Hermitian optical MEMS metasurfaces

**DOI:** 10.1126/sciadv.adl4661

**Published:** 2024-02-02

**Authors:** Fei Ding, Yadong Deng, Chao Meng, Paul C. V. Thrane, Sergey I. Bozhevolnyi

**Affiliations:** ^1^ Centre for Nano Optics, University of Southern Denmark, Campusvej 55, Odense DK-5230, Denmark.; ^2^ SINTEF Microsystems and Nanotechnology, Gaustadalleen 23C, 0737 Oslo, Norway.

## Abstract

Exceptional points (EPs), unique junctures in non-Hermitian open systems where eigenvalues and eigenstates simultaneously coalesce, have gained notable attention in photonics because of their enthralling physical principles and unique properties. Nonetheless, the experimental observation of EPs, particularly within the optical domain, has proven rather challenging because of the grueling demand for precise and comprehensive control over the parameter space, further compounded by the necessity for dynamic tunability. Here, we demonstrate the occurrence of optical EPs when operating with an electrically tunable non-Hermitian metasurface platform that synergizes chiral metasurfaces with piezoelectric MEMS mirrors. Moreover, we show that, with a carefully constructed metasurface, a voltage-controlled spectral space can be finely tuned to access not only the chiral EP but also the diabolic point characterized by degenerate eigenvalues and orthogonal eigenstates, thereby allowing for dynamic topological phase transition. Our work paves the way for developing cutting-edge optical devices rooted in EP physics and opening uncharted vistas in dynamic topological photonics.

## Teaser

The occurrence of optical exceptional points has been demonstrated with electrically tunable non-Hermitian MEMS metasurfaces.

## INTRODUCTION

In the realm of photonics, the exploration of exceptional points (EPs) has emerged as a fascinating avenue of research promising to reshape the landscape of light-matter interactions and optical manipulation (*1*–*5*). Rooted within the intricacies of non-Hermitian systems (*6*, *7*), EPs signify a unique configuration where distinct eigenvalues and eigenstates simultaneously coalesce, resulting in complex optical responses and enabling novel functionalities, including unidirectional transmission/reflection (*8*, *9*), exceptional sensing (*10*, *11*), asymmetric mode switching (*12*–*14*), and topological phase engineering (*15*). Recent scholarly attention within EP research has been directed toward non-Hermitian metasurfaces (*11*, *15*–*20*), a captivating platform that reveals the potential for implementing adaptive and precisely controlled optical functionalities through careful engineering of metasurface constituents’ geometrical and material attributes (*21*–*24*).

Nonetheless, existing experimental endeavors have predominantly been centered on passive metasurfaces, where the EP observation necessitates the fabrication of numerous samples, each featuring varying geometrical parameters and thus inadvertently adding fabrication errors. In addition, this method faces serious challenges in precisely attaining an EP, as unavoidable fabrication and measurement errors are amplified in the EP vicinity. In contrast, the emergence of dynamic, actively controlled metasurfaces indicates an alternative that would lead toward real-time control over the parameter space, facilitating the EP access and monitoring of the evolution of light-matter interactions near EPs within a single device (*25*, *26*). While certain advances along this route have been demonstrated, the practical realization of actively controlled non-Hermitian metasurfaces remains confined to the terahertz domain (*25*, *26*). The experimental observation of dynamically tuned EPs in the optical regime has thus far remained elusive, as the transition of EP metasurfaces from long-wavelength ranges (e.g., the terahertz range) to the optical range is nontrivial, involving not only scaling the dimensions down to a few hundred nanometers but also accounting for strongly dispersive optical constants, not to mention their dynamic tunability.

Here, we report on an in-depth exploration of optical EPs within a fully electrically tunable non-Hermitian metasurface platform that beneficially exploit the synergistic interplay between chiral metasurfaces and piezoelectric micro-electromechanical systems (MEMS) mirrors, thereby allowing for fine-tuning the system to construct a voltage-controlled spectral space containing not only the chiral EP but also the diabolic point (DP) characterized by degenerate eigenvalues and orthogonal eigenstates. By capitalizing on design flexibility and dynamic tunability, we demonstrate a voltage-controlled topological phase transition between EPs and DPs, representing a unique feature of the developed MEMS-based metasurface platform. Leveraging the dynamic attributes inherent to non-Hermitian optical metasurfaces, we endeavor initiating a new chapter characterized by fascinating prospects of enhanced light-matter interactions and advent of innovative EP-based optical devices.

## RESULTS

### Design of non-Hermitian metasurfaces for tunable topological phase transition

**Fig. 1.**
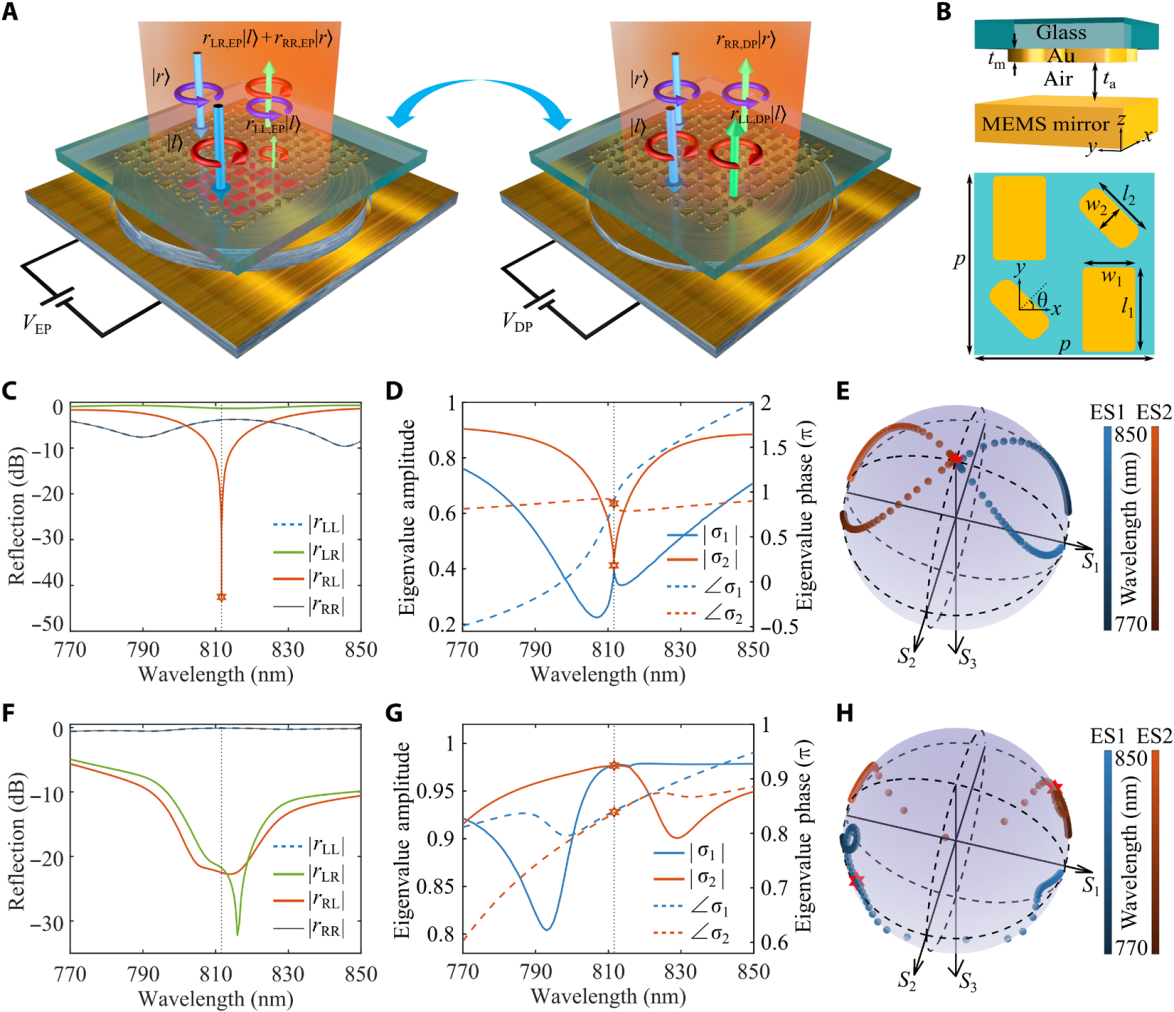
Design principle of the electrically tunable topological phase transition in non-Hermitian metasurfaces. (**A**) Schematic rendering of the tunable non-Hermitian metasurface composed of a chiral gold meta-atom array separated by a MEMS gold mirror with a voltage-controlled air gap to reconfigure the topological transition between a chiral EP and a DP. Here, the change in the propagation direction has not been considered in determining the chirality of reflected light. (**B**) Schematic illustration of a chiral unit cell. The geometric dimensions of the unit cell are set to *l*_1_ = 231.15 nm, *w*_1_ = 145.7 nm, *l*_2_ = 183.5 nm, *w*_2_ = 81.55 nm, θ = 45°, *p* = 500 nm, and *t*_m_ = 50 nm. The corners of small and large nanobricks are rounded with radii of 30 and 15 nm, respectively. (**C** to **E**) Simulated coefficients (C), eigenvalues (D), and eigenstates (E) of the reflection matrix as a function of wavelength at an air gap of *t*_a_ = 430.9 nm. The eigenvalues and eigenstates degenerate simultaneously at λ = 811.622 nm, indicating the existence of a chiral EP, where *r*_RL_ = 0 and an LCP state ∣*l*⟩ becomes the only eigenstate. (**F** to **H**) Simulated coefficients (F), eigenvalues (G), and eigenstates (H) of the reflection matrix as a function of wavelength at *t*_a_ = 357 nm. The two eigenvalues degenerate, while the two eigenstates are nearly orthogonal at λ = 811.622 nm, indicating the existence of a DP.

The electrically tunable non-Hermitian metasurface consists of a two-dimensional symmetry-broken chiral gold meta-atom array on a glass substrate and a thin-film piezoelectric MEMS mirror, where the air gap between the meta-atoms and MEMS mirror can be precisely controlled by applying an actuation voltage (Fig. 1A). For large air gaps, the near-field coupling between the gold meta-atoms and MEMS mirror disappears, switching the gap-surface plasmon resonance to a hybrid plasmonic Fabry-Pérot (FP) resonance that affords effective and dynamic control over reflected optical fields (*27*–*29*). The moveable MEMS mirror can vary the cavity length and, hence, tune the resonant wavelength, thereby modulating the coupling strength between the plasmonic and FP resonances. Meanwhile, a tunable far-field radiation rate (i.e., radiation loss) can be achieved with such a dynamic non-Hermitian metasurface (*30*). Capitalizing on the interplay between the plasmon and FP resonances via a voltage-controlled air gap, one could realize an electrically controlled topology transition.

The effective Hamiltonian of our dynamic metasurface can be described by a non-Hermitian Jones matrix $\hat{r}\left(\Omega \right)=\left[\begin{array}{cc} r_{\text{LL}}\left(\Omega \right) & r_{\text{LR}}\left(\Omega \right)\\ r_{\text{RL}}\left(\Omega \right) & r_{\text{RR}}\left(\Omega \right) \end{array}\right]$ , where *rij* is the reflection coefficient from *j*-polarized incident light to *i*-polarized reflected light in the circular polarization base, the subscript L or R represents left-handed or right-handed circularly polarized (LCP or RCP) light, and Ω = [*l*_1_, *w*_1_, *l*_2_, *w*_2_, *t*_a_(*V*_m_), λ] is the system parameter space shown in Fig. 1B. Because of the reciprocity and anisotropy, we have *r*_LL_(Ω) = *r*_RR_(Ω) and *r*_LR_(Ω) ≠ *r*_LR_(Ω). By adjusting the meta-atom properties and tuning the air gap with a proper actuation voltage *V*_EP_ (Supplementary Text and fig. S1), a singularity point has been observed in the parameter space of Ω_EP_ = [231.15, 145.7, 183.5, 81.55, 430.9, and 811.622 nm] (Fig. 1C), with the degeneracy of both eigenvalues and eigenstates simultaneously (Fig. 1D), indicating the existence of a chiral EP, where an LCP state ∣*L*⟩ becomes the only eigenstate due to coalescence (Fig. 1E). This degeneracy was further proven by the self-interacting Riemann surfaces of the reflection matrix eigenvalues in the geometrical parameter space (figs. S2 and S3). At the chiral EP, the reflection matrix becomes $\hat{r}\left(\Omega_{\text{EP}}\right)=\left(\begin{array}{cc} r_{\text{LL},\text{EP}} & r_{\text{LR},\text{EP}}\\ 0 & r_{\text{LL},\text{EP}} \end{array}\right)$ with eigenvalues coalescing into σ_1,2_ = *r*_LL,EP_, leading to the singular behavior in the reflection of light from the non-Hermitian metasurfaces. For LCP incidence (∣*E*_i_⟩ = ∣*l*⟩), the reflected light is maintained co-polarized (∣*E*_o_⟩ = *r*_LL,EP_∣*l*⟩), while the circular polarization conversion from LCP to RCP is prohibited. On the contrary, the reflected light superposes both LCP and RCP components (∣*E*_o_⟩ = *r*_LR,EP_∣*l*⟩ + *r*_RR,EP_∣*r*⟩) under the RCP excitation (∣*E*_i_⟩ = ∣*r*⟩). In a specific case where the incident polarization state equals to $\left|E_{i}\right\rangle =\left|l\right\rangle -\frac{r_{\text{LL},\text{EP}}}{r_{\text{LR},\text{EP}}}\left|r\right\rangle$ , two reflected LCP components destructively interfere and leave all incident waves converted to the RCP state ( $\left|E_{o}\right\rangle =-\frac{r_{\text{LL},\text{EP}}^{2}}{r_{\text{LR},\text{EP}}}\left|r\right\rangle$ ), which is orthogonal to the degenerated polarization eigenstate ∣*l*⟩ and indicates the reduced polarization eigenspace dimensionality at the chiral EP singularity. If co-polarized components are suppressed with *r*_LL,EP_ approaching 0, the non-Hermitian metasurface acts as a perfect LCP light absorber while reflecting the RCP light to its cross-polarized counterpart, enabling large circular dichroism (CD). Once the applied voltage *V*_m_ is varied from VEP, the length of the FP cavity and its resonance wavelength are finely tuned by moving the piezoelectric MEMS mirror with respect to the chiral gold meta-atom array, which switches the non-Hermitian metasurface away from the chiral EP and induces a topological phase transition. In particular, a DP was realized at an air gap of *t*_a_ = 357 nm (voltage *V*_DP_) when the chiral meta-atoms are located right at the nodes of the standing wave inside the non-Hermitian metasurface, where cross-polarized reflections vanish (Fig. 1F) and eigenvalues coalesce (Fig. 1G), while their associated eigenstates are orthogonal (Fig. 1H). Because of the nonperfect conducting nature of the gold mirror, the DP has complex eigenvalues, which is different from DP degeneracies in conservative Hermitian systems that have real eigenvalues. At the DP, the effective Hamiltonian changes to a diagonal reflection matrix $\hat{r}\left(\Omega_{\text{DP}}\right)=\left(\begin{array}{cc} r_{\text{LL},\text{DP}} & 0\\ 0 & r_{\text{LL},\text{DP}} \end{array}\right)$ with eigenvalues coalescing into σ_1,2_ = *r*_LL,DP_ and two polarization eigenstates being orthogonally aligned, thereby allowing simultaneous nulling of cross-polarized reflections under both LCP and RCP incidence (Fig. 1, A and F).

### Experimental observation of the dynamic topological transition

**Fig. 2.**
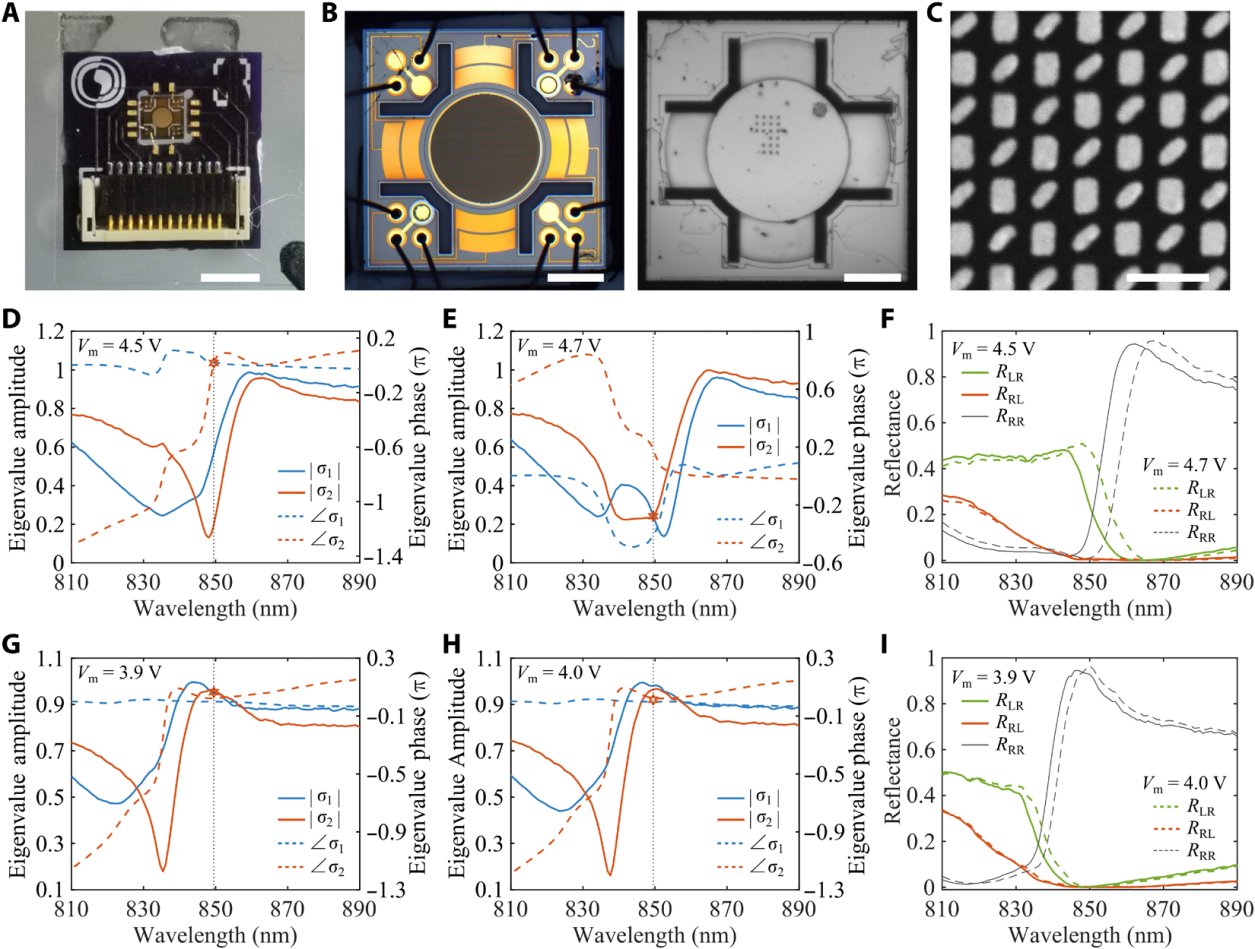
Experimental observation of the dynamic topological transition from a chiral EP to a DP. (**A** to **C**) The typical photo (A), optical microscopy images (B), and scanning electron microscopy image (C) of the assembled non-Hermitian metasurface. Scale bars, 3 mm (A), 500 μm (B), and 500 nm (C). (**D** to **F**) Measured eigenvalues [(D) and (E)] and reflectance (F) as a function of wavelength at two different voltages of *V*_m_ = 4.5 and 4.7 V when driving four outer electrodes. Anti-crossing of eigenvalue amplitudes and crossing of eigenvalue phases are observed for *V*_m_ = 4.5 V, while crossing of eigenvalue amplitudes and anti-crossing of eigenvalue phases are observed for *V*_m_ = 4.7 V, revealing a chiral EP singularity at λ = 849.505 nm for *V*_m_ between 4.5 and 4.7 V. (**G** to **I**) Measured eigenvalues [(G) and (H)] and reflectance (I) as a function of wavelength at two different voltages of *V*_m_ = 3.9 and 4.0 V when driving four outer electrodes. Crossing of eigenvalue amplitudes is observed for *V*_m_ = 3.9 V, indicating a DP at λ = 849.505 nm for *V*_m_ of ~3.9 V.

To experimentally observe the topological phase transition between EPs and DPs, the dynamic non-Hermitian metasurface was fabricated by assembling a chiral gold meta-atom array, a ultraflat MEMS gold mirror, and a printed circuit board via wire bonding (see Materials and Methods and Fig. 2, A to C), forming a hybrid FP cavity with a variable air gap *t*_a_, which is precisely controlled by electrically activating the MEMS mirror as a moveable back reflector (*27*–*29*). For the optical characterization of the fabricated non-Hermitian metasurface, we used a custom-built optical setup that consists of broadband laser sources and different optical components for polarization-resolved imaging and detection (see Materials and Methods and fig. S4). In the measurement, the MEMS metasurface was triggered with an actuation voltage *V*_m_ in a step of 0.1 V, corresponding to an averaged air gap moving step of ~7.3 nm, which allows us to finely detune the non-Hermitian metasurface to construct voltage-controlled parameter space. The moveable range of the assembled metasurface was estimated to be ~868.6 nm (~730.1 nm) when the MEMS mirror was moved far away from (close to) the chiral meta-atom array by selectively actuating four outer (inner) electrodes from 0 to 12 V (0 to 10 V) (fig. S5), which covers at least two adjacent FP resonances and is enough for investigating the topological phase transition. From the measured reflection spectra at each voltage (figs. S6 and S7), the wavelength-resolved Stokes parameters along with the non-Hermitian Jones matrix $\hat{r}$ can be obtained (see Materials and Methods). At a voltage of *V*_m_ = 4.5 V (*t*_a_ ≈ 2534.6 nm), we observe an anti-crossing of eigenvalue phases and a crossing of eigenvalue amplitudes λ = 849.505 nm (Fig. 2D). As the actuation voltage varies, the coupling between plasmonic and FP resonances is tuned, leading to the interchange in the crossing behavior of amplitudes and phases. When *V*_m_ is decreased to 4.7 V (*t*_a_ = 2546.8 nm), we see crossing in amplitudes and anti-crossing in phases (Fig. 2E). Therefore, a chiral EP singularity unambiguously occurs at λ = 849.505 nm for *V*_m_ between 4.5 and 4.7 V (*11*, *17*) where singularity points are observed for *R*_RL_ (Fig. 2F), although the experimental wavelength and air gap are deviated from simulated results due to the imperfections in fabrication. Owing to the FP nature of the non-Hermitian metasurface, the chiral EP singularity can be periodically observed at other air gaps (fig. S8). Given the high sensitivity of non-Hermitian systems near EPs, we detuned the MEMS metasurface to demonstrate a non-Hermitian phase transition of a chiral EP singularity to a DP by carefully altering the air gap with an actuation voltage. When the voltage is continuously varied from 4.7 to 3.9 V, corresponding to the air gap decrease from 2546.8 to 2487.1 nm, the non-Hermitian metasurface gradually transits from an EP to a DP with a crossing of eigenvalue amplitudes at the wavelength of λ = 849.505 nm (Fig. 2G). For a slightly increased voltage of *V*_m_ = 4.0 V (*t*_a_ = 2496.4 nm), the phases of two eigenvalues approach a crossing state (Fig. 2H). Different from the EPs that are extremely sensitive to small perturbations (*10*, *11*), non-Hermitian DPs are more robust with eigenvalues slightly affected by the voltage. Meanwhile, simultaneous nulling of both cross-polarized reflections in the vicinity of the DP under RCP and LCP incidence is allowed (Fig. 2I). It is worth noting that another pair of exceptional and diabolic points that enable the similar topological phase transition was observed in the [*t*_a_(*V*_m_), λ] parameter space without repeated fabrication of several samples (figs. S9 and S10), which is ascribed to the tunability of our non-Hermitian MEMS metasurface.

### Voltage-controlled polarization evolution

**Fig. 3.**
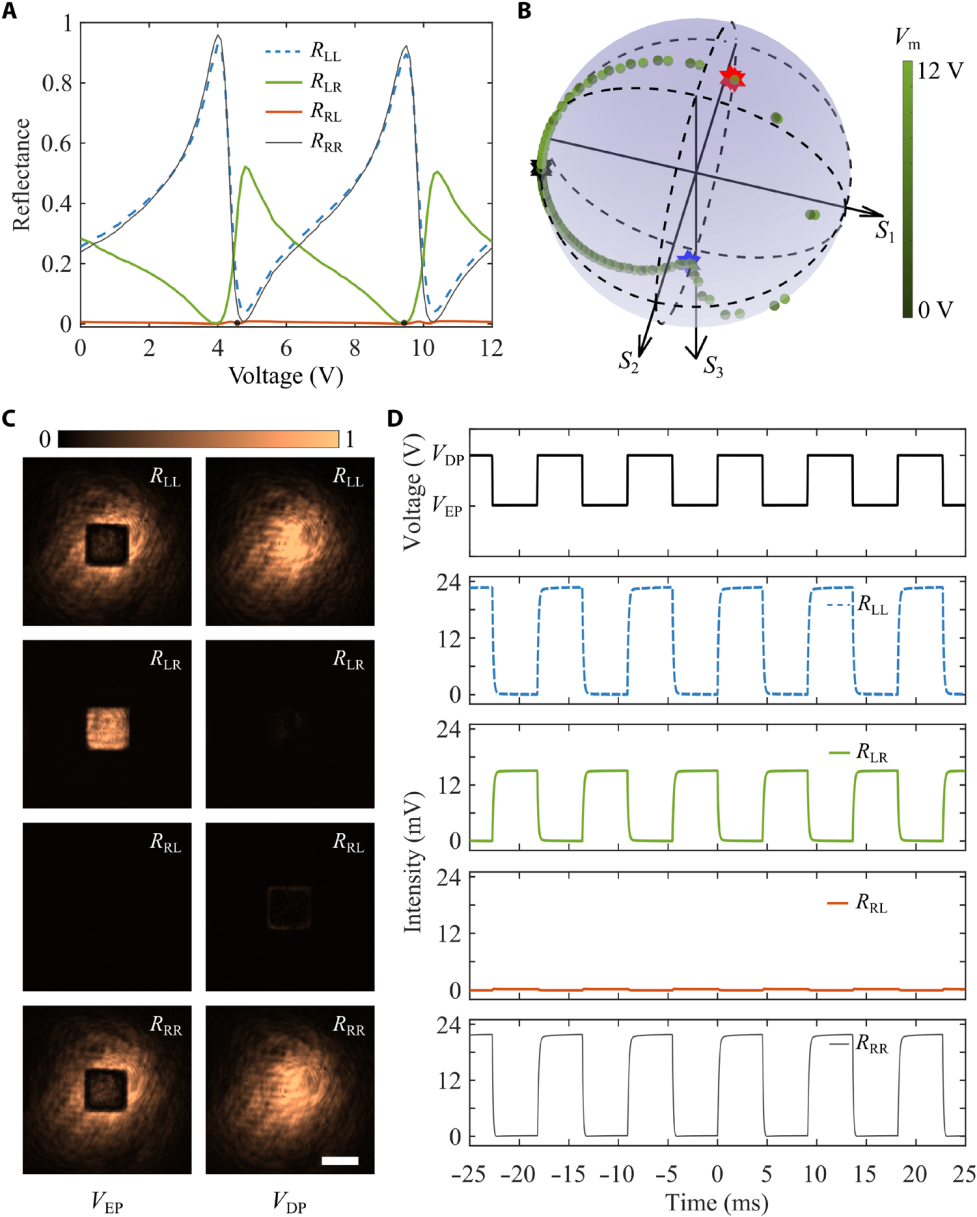
Voltage-controlled polarization evolution. (**A**) Measured reflectance as a function of the applied voltage. (**B**) Voltage-controlled polarization trajectory mapped on the Poincaré sphere for RCP incidence. (**C**) Polarization-resolved optical images of the reflected light for the topological EP-DP transition. Scale bar, 25 μm. (**D**) Temporal evolution of the reflected power for the topological EP-DP transition by actuating the non-Hermitian metasurface with a periodic rectangular voltage. The rise/fall times of the *R*_LL_, *R*_LR_, and *R*_RR_ channels are 260/250 μs, 290/220 μs, and 260/160 μs, respectively. The wavelength is fixed at λ = 849.505 nm, and the four outer electrodes are actuated during the measurement.

In addition to the dynamic non-Hermitian phase transition, we also explored voltage-controlled polarization evolution when the air gap is continuously varied. For LCP incidence at λ = 849.505 nm, the cross-polarized reflection approaches zero regardless of the actuation voltage (Fig. 3A). Once the incident light is switched to RCP, the output polarization state becomes a superposition of co- and cross-polarized components, whose intensities are electrically modulated (Fig. 3A). Moreover, its corresponding polarization trajectory is one irregular closed curve on the Poincaré sphere, passing through two pivotal polarization states of ∣*l*⟩ and ∣*r*⟩ when the non-Hermitian metasurface hits the exceptional and diabolic points, and circulating repeatedly with the voltage (Fig. 3B). Quantitatively, the degrees of circular polarization (DoCPs) reach ~−0.96 and ~0.99 at EPs and DPs, respectively (red and blue stars in Fig. 3B). At chiral EPs, asymmetric polarization conversion with a measured CD of ~0.49 was obtained, resulting from the reduced polarization eigenspace. Besides circularly polarized (CP) waves, linearly polarized reflections of high purity (DoCP < 0.02) were realized (black stars in Fig. 3B). Note that the minimum voltage range to electrically switch the reflected light between ∣*l*⟩ and ∣*r*⟩ states is only 0.8 V, which is smaller than the previously required value of 4.7 V (*28*). Because the MEMS arrangements used in both experiments are identical, such a strong decrease in the switching voltage signifies a marked decrease (by ~34.5 times) in the switching power, thereby manifesting a very substantial achievement resulting from the operation near the EP-DP transition. As expected, a similar polarization evolution is seen when the four inner electrodes are actuated to decrease the air gap (fig. S11). To measure the non-Hermitian phase transition speed from an EP to a DP, we actuated the MEMS mirror with a periodic rectangular signal composed of alternating voltages marked in Fig. 3A and detected polarization-resolved reflections with a fast photodetector (see Materials and Methods). We observe high contrast between orthogonal polarization states (Fig. 3C), good endurance (Fig. 3D and movies S1 and S2), and relatively fast switching with rise/fall times of ~0.30/0.25 ms (Fig. 3D). Because response times are generally dependent on the properties of MEMS mirrors, one could expect faster switching even in the megahertz range via further optimization, which, however, is beyond the scope of our work.

## DISCUSSION

In this work, we have demonstrated an electrically tunable non-Hermitian MEMS metasurface to investigate optical chiral EPs in a voltage-controlled spectral domain through meticulous system detuning. By carefully actuating the air gap between the chiral metasurface and MEMS mirror, we have realized a fast (<0.30 ms) voltage-driven topological phase transition between EPs and DPs. Notably, our implementation of the non-Hermitian MEMS metasurface facilitates the robust and dynamic polarization conversion between two circular polarization states (i.e., ∣*l*⟩ and ∣*r*⟩) with an extremely small voltage step of 0.8 V. Furthermore, compared to other tunable optical metasurfaces that use active materials with reconfigurable properties to directly build meta-atoms (*31*–*34*), our non-Hermitian MEMS metasurface vertically integrates the functional metasurface with moveable MEMS mirror and is thus not limited by the submicrometer-thin planar configuration, thereby exhibiting considerably large tunability ranges for achieving the EP-DP phase transition in a fast and reversible fashion. Our results not only pave the way for the development of cutting-edge EP-empowered optical devices at small scales [e.g., adaptive polarization optics for biomedical applications (*35*) and advanced sensors by combining the EP and DP operation modes (fig. S12)] but also unveil unexplored avenues within the dynamic landscape of topological photonics.

## MATERIALS AND METHODS

### Numerical simulations

All numerical simulations were performed using the wave optics module in COMSOL Multiphysics version 5.6, where we modeled one glass-gold-air-gold unit cell. The corners of small and large nanobricks are rounded with radii of 30 and 15 nm, respectively. Periodic boundary conditions were applied in both the *x* and *y* directions, and perfectly matched layers were used in the *z* direction to truncate the simulation domain. To obtain complex reflection coefficients in both linear and circular polarization bases, *x*- and *y*-polarized light sources were incident onto the chiral gold meta-atom from the upper glass at normal incidence. The glass layer was regarded as a lossless dielectric with a constant refractive index of 1.46, and the permittivity of gold was interpolated from experimental values (*36*).

### Device fabrication

The chiral gold meta-atoms were fabricated using electron beam lithography (EBL), thin-film deposition, and lift-off techniques. First, a 100-nm-thick poly(methyl methacrylate) (2% in anisole, Micro Chem) layer and a 40-nm-thick conductive polymer layer (AR-PC 5090, Allresist) were successively spin-coated on a glass substrate (Borofloat 33 wafer, Wafer Universe). Then, the metasurface patterns were defined in the center area of the glass substrate using EBL (JEOL JEM-6500F) at an acceleration voltage of 30 kV. After exposure, the sample was soaked in deionized water for 1 min to remove the conductive polymer and subsequently developed in the solution of methyl isobutyl ketone (MIBK) and isopropyl alcohol (IPA) of MIBK:IPA = 1:3 for 35 s followed by 60 s in an IPA bath. After that, a 1-nm titanium adhesion layer and a 50-nm gold layer were deposited using thermal evaporation (Tornado 400, Cryofox). Last, the gold structures were formed atop the glass substrate after a liftoff process in acetone. The MEMS mirror is fabricated using standard semiconductor manufacturing processes, where thin-film lead zirconate titanate is incorporated to achieve enduring and low-voltage electrical actuation (*27*–*29*). After depositing a 100-nm-thick gold layer to function as a back reflector, the MEMS mirror surface is inspected using white light interferometry (Zygo NewView 6000) to guarantee good flatness over the whole MEMS mirror with a diameter of ~1 mm. The non-Hermitian MEMS metasurface was assembled by gluing the glass substrates with prefabricated chiral meta-atoms and the selected clean MEMS mirror. Last, the assembled metasurface was glued to a printed circuit board, followed by gold wire bonding for electrical connection and actuation.

### Optical characterization

Figure S4 shows the optical setup for characterizing non-Hermitian MEMS metasurfaces. A fiber-coupled supercontinuum laser (SuperK Extreme, NKT Photonics) passes through a collimator (TC06APC-780, Thorlabs), a half-wave plate (AHWP10M-980, Thorlabs), an attenuator (NE01B, Thorlabs), a sliver mirror (PF10-03-P01, Thorlabs), a linear polarizer (LP1, LPNIR050-MP2, Thorlabs), a quarter-wave plate (QWP1; AQWP10M-980, Thorlabs) mounted on a motorized precision rotation stage (PRM1Z8, Thorlabs), and two beam splitters (BS1 and BS2, CCM1-BS014/M, Thorlabs) successively to create CP light with controlled intensity. The two beam splitters could compensate for the polarization-dependent phase shifts caused by one single beam splitter. The CP light is then slightly focused onto the sample by using a long working distance objective (Objective M Plan Apo 20×/0.42 numeric aperture, Mitutoyo). The reflected light is collected by the same objective and passes through BS2, a flip quarter-wave plate (QWP2; AQWP10M-980, Thorlabs), and a tube lens (*f* = 200 mm; TTL200-S8, Thorlabs), generating the first real image plane where an iris (SM1D12SZ, Thorlabs) is positioned for filtering out the background outside the sample area (scale bar is 10 μm). The filtered first real image is transformed by a relay lens (*f* = 200 mm; AC254-200-B-ML, Thorlabs) to create a Fourier image, which is captured by a complementary metal oxide semiconductor camera (DCC1545M, Thorlabs) when a flip mirror (PF10-03-P01, Thorlabs) is flipped up. To switch between real and Fourier images, a flip lens (*f* = 100 mm; AC254-100-B-ML, Thorlabs) is placed between the relay lens and the flip mirror. For full Stokes polarimetry, a Stokes analyzer composed of QWP2, a linear polarizer (LP2; LPNIR050-MP2, Thorlabs), and a spectrometer (QE Pro, Ocean Optics) is used.

In the measurement, we used a fiber-coupled spectrometer to record polarization-resolved spectra of *I*_x_(λ), *I*_y_(λ), *I*_a_(λ), *I*_b_(λ), *I*_r_(λ), and *I*_l_(λ) under the RCP and LCP incident light. We also recorded the reflection spectrum from the MEMS gold mirror *I*_sub_(λ). The Stokes parameters (*s*_1_, *s*_2_, and *s*_3_) without any normalization can be calculated as

$$s_{1}\left(\lambda \right)=\left[I_{x}\left(\lambda \right)-I_{y}\left(\lambda \right)\right]/I_{\text{sub}}\left(\lambda \right)\times T_{\text{QWP}}\left(\lambda \right)$$ (1)

$$s_{2}\left(\lambda \right)=[I_{a}\left(\lambda \right)-I_{b}\left(\lambda \right)]/I_{\text{sub}}\left(\lambda \right)\times T_{\text{QWP}}\left(\lambda \right)$$ (2)

$$s_{3}\left(\lambda \right)=\left[I_{r}\left(\lambda \right)-I_{l}\left(\lambda \right)\right]/I_{\text{sub}}\left(\lambda \right)$$ (3)

where *T*_QWP_(λ) is the measured transmittance of the QWP. The reflected light $\left[\begin{array}{c} E_{x}\left(\lambda \right)\\ E_{y}\left(\lambda \right) \end{array}\right]$ in the linear polarization base can be retrieved as

$$E_{x}\left(\lambda \right)=\sqrt{\frac{s_{1}\left(\lambda \right)+\sqrt{s_{1}{\left(\lambda \right)}^{2}+s_{2}{\left(\lambda \right)}^{2}+s_{3}{\left(\lambda \right)}^{2}}}{2}}$$ (4)

$$E_{y}\left(\lambda \right)=\sqrt{\frac{-s_{1}\left(\lambda \right)+\sqrt{s_{1}{\left(\lambda \right)}^{2}+s_{2}{\left(\lambda \right)}^{2}+s_{3}{\left(\lambda \right)}^{2}}}{2}}e^{i∙\text{atan}\frac{s_{3}\left(\lambda ,\right)}{s_{2}\left(\lambda \right)}}$$ (5)

Then, the reflected light in the circular polarization base can be written as

$$\left[\begin{array}{c} E_{l}\left(\lambda \right)\\ E_{r}\left(\lambda \right) \end{array}\right]=\frac{\sqrt{2}}{2}\left(\begin{array}{cc} 1 & -i\\ 1 & i \end{array}\right)\left[\begin{array}{c} E_{x}\left(\lambda \right)\\ E_{y}\left(\lambda \right) \end{array}\right]$$ (6)

Last, the measured non-Hermitian Jones matrix $\hat{r}\left(\lambda \right)=\left[\begin{array}{cc} r_{\text{LL}}\left(\lambda \right) & r_{\text{LR}}\left(\lambda \right)\\ r_{\text{RL}}\left(\lambda \right) & r_{\text{RR}}\left(\lambda \right) \end{array}\right]$ can be obtained.

To measure the response time of the phase transition between exceptional and diabolic points, the experimental setup in fig. S4 is modified by replacing the input laser and spectrometer with a continuous wave Ti:sapphire laser (Spectra-Physics 3900 S) and a photodetector (PDA20CS-EC, Thorlabs), respectively. The signals from the photodetector are captured using an oscilloscope (DSOX2024A, Keysight). In the measurements, the non-Hermitian MEMS metasurface sample is actuated with periodically alternating voltages.

## References

[1] L. Feng, R. El-Ganainy, L. Ge, Non-Hermitian photonics based on parity-time symmetry. Nat. Photonics 11, 752–762 (2017).

[2] R. El-Ganainy, K. G. Makris, M. Khajavikhan, Z. H. Musslimani, S. Rotter, D. N. Christodoulides, Non-Hermitian physics and PT symmetry. Nat. Phys. 14, 11–19 (2018).

[3] Ş. K. Özdemir, S. Rotter, F. Nori, L. Yang, Parity-time symmetry and exceptional points in photonics. Nat. Mater. 18, 783–798 (2019). 30962555 10.1038/s41563-019-0304-9

[4] M.-A. Miri, A. Alù, Exceptional points in optics and photonics. Science 363, eaar7709 (2019). 30606818 10.1126/science.aar7709

[5] A. Li, H. Wei, M. Cotrufo, W. Chen, S. Mann, X. Ni, B. Xu, J. Chen, J. Wang, S. Fan, C.-W. Qiu, A. Alù, L. Chen, Exceptional points and non-Hermitian photonics at the nanoscale. Nat. Nanotechnol. 18, 706–720 (2023). 37386141 10.1038/s41565-023-01408-0

[6] C. M. Bender, S. Boettcher, Real spectra in non-hermitian hamiltonians having *PT* symmetry. Phys. Rev. Lett. 80, 5243–5246 (1998).

[7] C. M. Bender, Making sense of non-Hermitian Hamiltonians. Rep. Prog. Phys. 70, 947–1018 (2007).

[8] Z. Lin, H. Ramezani, T. Eichelkraut, T. Kottos, H. Cao, D. N. Christodoulides, Unidirectional invisibility induced by *PT*-symmetric periodic structures. Phys. Rev. Lett. 106, 213901 (2011). 21699297 10.1103/PhysRevLett.106.213901

[9] L. Feng, Y. L. Xu, W. S. Fegadolli, M. H. Lu, J. E. B. Oliveira, V. R. Almeida, Y. F. Chen, A. Scherer, Experimental demonstration of a unidirectional reflectionless parity-time metamaterial at optical frequencies. Nat. Mater. 12, 108–113 (2013). 23178268 10.1038/nmat3495

[10] W. J. Chen, S. K. Ozdemir, G. M. Zhao, J. Wiersig, L. Yang, Exceptional points enhance sensing in an optical microcavity. Nature 548, 192–196 (2017). 28796206 10.1038/nature23281

[11] J. H. Park, A. Ndao, W. Cai, L. Hsu, A. Kodigala, T. Lepetit, Y.-H. Lo, B. Kanté, Symmetry-breaking-induced plasmonic exceptional points and nanoscale sensing. Nat. Phys. 16, 462–468 (2020).

[12] J. Doppler, A. A. Mailybaev, J. Böhm, U. Kuhl, A. Girschik, F. Libisch, T. J. Milburn, P. Rabl, N. Moiseyev, S. Rotter, Dynamically encircling an exceptional point for asymmetric mode switching. Nature 537, 76–79 (2016). 27454554 10.1038/nature18605

[13] X. L. Zhang, T. Jiang, C. T. Chan, Dynamically encircling an exceptional point in anti-parity-time symmetric systems: Asymmetric mode switching for symmetry-broken modes. Light Sci. Appl. 8, 88 (2019). 31645932 10.1038/s41377-019-0200-8 PMC6804564

[14] A. Schumer, Y. Liu, J. Leshin, L. Ding, Y. Alahmadi, A. Hassan, H. Nasari, S. Rotter, D. Christodoulides, P. LiKamWa, M. Khajavikhan, Topological modes in a laser cavity through exceptional state transfer. Science 375, 884–888 (2022). 35201888 10.1126/science.abl6571

[15] Q. Song, M. Odeh, J. Zúñiga-Pérez, B. Kanté, P. Genevet, Plasmonic topological metasurface by encircling an exceptional point. Science 373, 1133–1137 (2021). 34516834 10.1126/science.abj3179

[16] M. Lawrence, N. Xu, X. Zhang, L. Cong, J. Han, W. Zhang, S. Zhang, Manifestation of *PT* symmetry breaking in polarization space with terahertz metasurfaces. Phys. Rev. Lett. 113, 093901 (2014). 25215984 10.1103/PhysRevLett.113.093901

[17] S. H. Park, S.-G. Lee, S. Baek, T. Ha, S. Lee, B. Min, S. Zhang, M. Lawrence, T.-T. Kim, Observation of an exceptional point in a non-Hermitian metasurface. Nanophotonics 9, 1031–1039 (2020).

[18] Z. Li, G. Cao, C. Li, S. Dong, Y. Deng, X. Liu, J. S. Ho, C.-W. Qiu, Non-Hermitian electromagnetic metasurfaces at exceptional points (Invited Review). Prog. Electromagn. Res. 171, 1–20 (2021).

[19] J. Yu, B. Ma, A. Ouyang, P. Ghosh, H. Luo, A. Pattanayak, Q. Li, Dielectric super-absorbing metasurfaces via PT symmetry breaking. Optica 8, 1290–1295 (2021).

[20] Y. Xu, L. Li, H. Jeong, S. Kim, I. Kim, J. Rho, Y. Liu, Subwavelength control of light transport at the exceptional point by non-Hermitian metagratings. Sci. Adv. 9, eadf3510 (2023). 37172089 10.1126/sciadv.adf3510 PMC10181182

[21] H. H. Hsiao, C. H. Chu, D. P. Tsai, Fundamentals and applications of metasurfaces. Small Methods 1, 1600064 (2017).

[22] F. Ding, A. Pors, S. I. Bozhevolnyi, Gradient metasurfaces: A review of fundamentals and applications. Rep. Prog. Phys. 81, 026401 (2018). 28825412 10.1088/1361-6633/aa8732

[23] S. Sun, Q. He, J. Hao, S. Xiao, L. Zhou, Electromagnetic metasurfaces: Physics and applications. Adv. Opt. Photonics 11, 380–479 (2019).

[24] A. M. Shaltout, V. M. Shalaev, M. L. Brongersma, Spatiotemporal light control with active metasurfaces. Science 364, eaat3100 (2019). 31097638 10.1126/science.aat3100

[25] M. S. Ergoktas, S. Soleymani, N. Kakenov, K. Wang, T. B. Smith, G. Bakan, S. Balci, A. Principi, K. S. Novoselov, S. K. Ozdemir, C. Kocabas, Topological engineering of terahertz light using electrically tunable exceptional point singularities. Science 376, 184–188 (2022). 35389774 10.1126/science.abn6528 PMC7612901

[26] S. Baek, S. H. Park, D. Oh, K. Lee, S. Lee, H. Lim, T. Ha, H. S. Park, S. Zhang, L. Yang, B. Min, T. T. Kim, Non-Hermitian chiral degeneracy of gated graphene metasurfaces. Light Sci. Appl. 12, 87 (2023). 37024464 10.1038/s41377-023-01121-6 PMC10079968

[27] C. Meng, P. C. Thrane, F. Ding, J. Gjessing, M. Thomaschewski, C. Wu, C. Dirdal, S. I. Bozhevolnyi, Dynamic piezoelectric MEMS-based optical metasurfaces. Sci. Adv. 7, eabg5639 (2021). 34162551 10.1126/sciadv.abg5639 PMC8221626

[28] C. Meng, P. C. Thrane, F. Ding, S. I. Bozhevolnyi, Full-range birefringence control with piezoelectric MEMS-based metasurfaces. Nat. Commun. 13, 2071 (2022). 35440591 10.1038/s41467-022-29798-0 PMC9018774

[29] P. C. V. Thrane, C. Meng, F. Ding, S. I. Bozhevolnyi, MEMS tunable metasurfaces based on gap plasmon or Fabry-Pérot resonances. Nano Lett. 22, 6951–6957 (2022). 35980825 10.1021/acs.nanolett.2c01692 PMC9479152

[30] R. Ameling, H. Giessen, Microcavity plasmonics: Strong coupling of photonic cavities and plasmons. Laser Photon. Rev. 7, 141–169 (2013).

[31] C. H. Chu, M. L. Tseng, J. Chen, P. C. Wu, Y. H. Chen, H. C. Wang, T. Y. Chen, W. T. Hsieh, H. J. Wu, G. Sun, D. P. Tsai, Active dielectric metasurface based on phase-change medium. Laser Photon. Rev. 10, 986–994 (2016).

[32] P. C. Wu, R. A. Pala, G. Kafaie Shirmanesh, W. H. Cheng, R. Sokhoyan, M. Grajower, M. Z. Alam, D. Lee, H. A. Atwater, Dynamic beam steering with all-dielectric electro-optic III–V multiple-quantum-well metasurfaces. Nat. Commun. 10, 3654 (2019). 31409790 10.1038/s41467-019-11598-8 PMC6692380

[33] G. Qu, W. Yang, Q. Song, Y. Liu, C.-W. Qiu, J. Han, D.-P. Tsai, S. Xiao, Reprogrammable meta-hologram for optical encryption. Nat. Commun. 11, 5484 (2020). 33127918 10.1038/s41467-020-19312-9 PMC7603497

[34] P. C. Wu, R. Sokhoyan, G. K. Shirmanesh, W. H. Cheng, H. A. Atwater, Near-infrared active metasurface for dynamic polarization conversion. Adv. Opt. Mater. 9, 2100230 (2021).

[35] C. He, H. He, J. Chang, B. Chen, H. Ma, M. J. Booth, Polarisation optics for biomedical and clinical applications: A review. Light Sci. Appl. 10, 194 (2021). 34552045 10.1038/s41377-021-00639-x PMC8458371

[36] P. B. Johnson, R. W. Christy, Optical constants of the noble metals. Phys. Rev. B 6, 4370–4379 (1972).

