# Supplementary material for: Electrically tunable topological phase transition in non-Hermitian optical MEMS metasurfaces
Source: Sci Adv. 2024 Feb 2;10(5):eadl4661. doi: 10.1126/sciadv.adl4661 (PMC10836917; doi:10.1126/sciadv.adl4661)
Supplement: Supplementary file 1 — Supplementary Text Figs. S1 to S12 Legends for movies S1 and S2 [file sciadv.adl4661_sm.pdf]

Science Advances
AAAS

Supplementary Materials for

Electrically tunable topological phase transition in non-Hermitian optical MEMS metasurfaces

Fei Ding *et al.*

Corresponding author: Fei Ding, feid@mci.sdu.dk; Sergey I. Bozhevolnyi, seib@mci.sdu.dk

Sci. Adv. **10**, eadl4661 (2024)
DOI: 10.1126/sciadv.adl4661

The PDF file includes:

Supplementary Text
Figs. S1 to S12
Legends for movies S1 and S2

Other Supplementary Material for this manuscript includes the following:

Movies S1 and S2

Supplementary Text

<u>Design of the non-Hermitian optical MEMS metasurface</u>

To design the non-Hermitian metasurface possessing a chiral exceptional point (EP), we first consider a glass-gold-air-gold building block with only one gold nanobrick that is not rotated (e.g., the period is 250 nm). After sweeping the lateral dimensions of L_x and L_y, we select two elements that function as half-wave plates (HWPs) with equal reflection amplitudes but an intrinsic phase difference of π/2 at the design wavelength of λ = 810 nm with an air gap of t_a = 430 nm (fig. S1, A and B). The Jones matrices of these two HWPs in the circular polarization base are given by $\widehat{r_1} = r_{xx1}\begin{pmatrix} 0 & 1 \\ 1 & 0 \end{pmatrix}$ and $\widehat{r_2} = r_{xx2}\begin{pmatrix} 0 & 1 \\ 1 & 0 \end{pmatrix} = r_{xx1}e^{-i\frac{\pi}{2}}\begin{pmatrix} 0 & 1 \\ 1 & 0 \end{pmatrix}$ respectively, where r_{xx1} and r_{xx2} are the co-polarized reflection coefficients in the linear polarization base under x-polarized excitation. The reflection matrix of HWP2 with a rotation angle of θ_0 = 45° is expressed as $\widehat{r_2}(\theta_0) = r_{xx1}\begin{pmatrix} 0 & 1 \\ -1 & 0 \end{pmatrix}$. By arranging these two HWPs in a unit cell with a period of p = 500 nm, the averaged Jones matrix can be approximately derived as $\hat{r} \approx \frac{1}{2}[\widehat{r_1} + \widehat{r_2}(\theta_0)] = r_{xx1}\begin{pmatrix} 0 & 1 \\ 0 & 0 \end{pmatrix}$, which shows the potential to design the chiral non-Hermitian metasurface. However, due to the near-field coupling between nanobricks within the designed unit cell, the metasurface does not show the expected performance at λ = 810 nm (fig. S1C). Therefore, the meta-atom dimensions, air gap, and wavelength should be iteratively optimized to realize chiral EP singularity. On the contrary, the diabolic point can still be observed near the wavelength of ~ 812 nm with the air gap of t_a = 357 nm (fig. S1D), where the metasurface is located right at the nodes of the standing wave with zero illumination intensity.

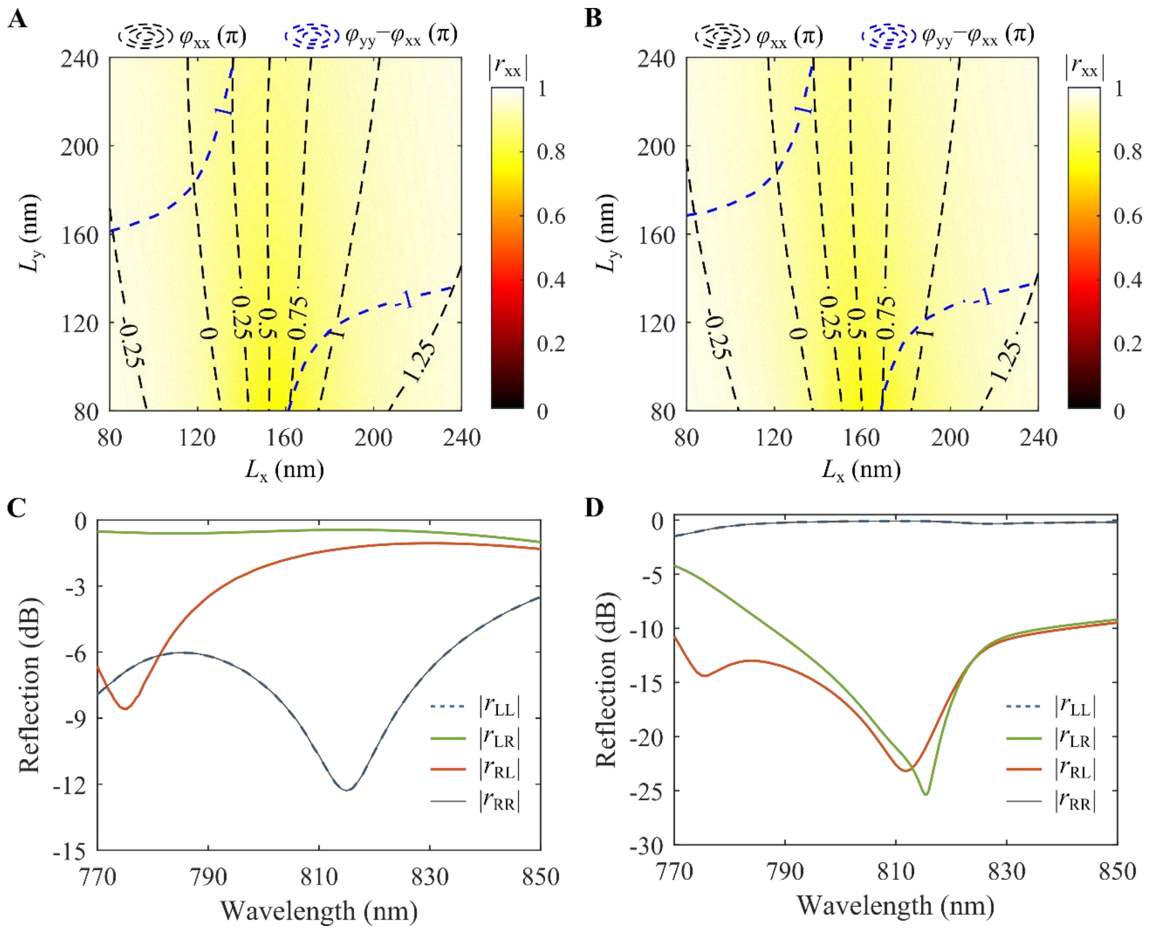

Fig. S1. Simulation of the as-designed non-Hermitian optical metasurfaces. (A and B) Simulated complex reflection coefficients of the glass-gold-air-gold building block with only one nanobrick (period is 250 nm) as a function of nanobrick dimensions of L_x and L_y in the linear polarization base. The other parameters are as follows: λ = 810 nm, t_m = 50 nm, and t_a = 430 nm. The nanobricks are rounded with radii of 15 nm (A) and 30 nm (B), respectively. $|r_{xx}|$ is the reflection amplitude under x-polarized excitation, and φ_{xx} and φ_{yy} represent the corresponding phase shifts under x- and y-polarized excitations. (C and D) Simulated coefficients of the reflection matrix as a function of wavelength at air gaps of t_a = 430 nm (C) and 357 nm (D). The geometric dimensions of the unit cell (same configuration as that in Fig. 1B) are set to l_1 = 232 nm, w_1 = 135 nm, l_2 = 168 nm, w_2 = 79 nm, θ = 45°, p = 500 nm, and t_m = 50 nm. The corners of small and large nanobricks are rounded with radii of 30 nm and 15 nm, respectively.

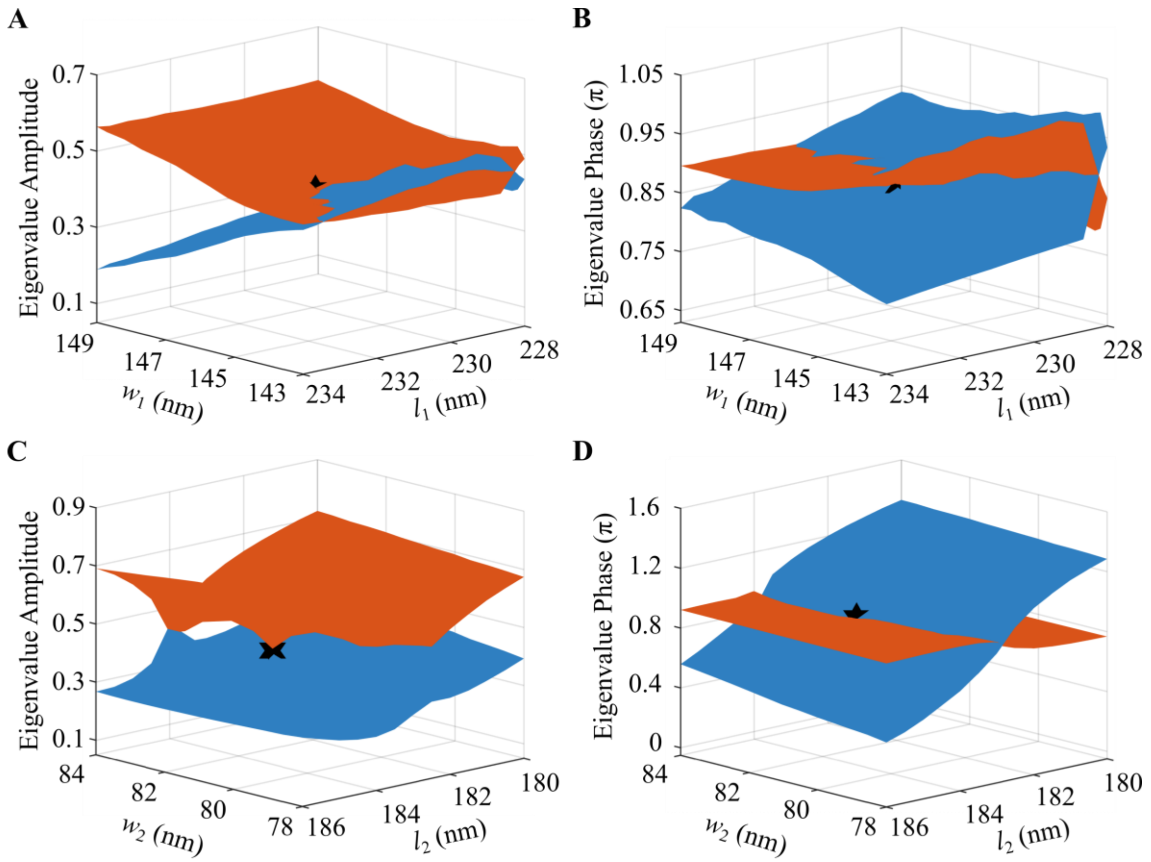

Fig. S2. Simulated amplitudes (A and C) and phase (B and D) of the reflection matrix eigenvalues in the geometrical parameter space $\Omega = [l_1, w_1]$ (A and B) and $[l_2, w_2]$ (C and D). Self-intersecting Riemann surfaces are observed. The wavelength and air gap are set as 811.622 nm and 430.9 nm, respectively.

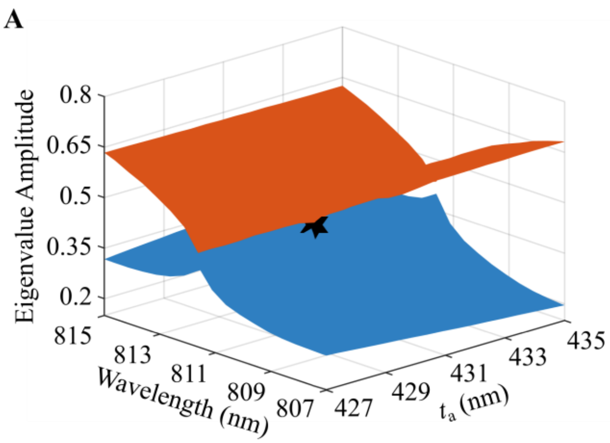

Fig. S3. Simulated amplitudes (A) and phase (B) of the reflection matrix eigenvalues in the geometrical parameter space $\boldsymbol{\Omega} = [\boldsymbol{t_a}, \boldsymbol{\lambda}]$. A self-intersecting Riemann surface is observed. The dimensions of the chiral meta-atom are the same as those in the main text.

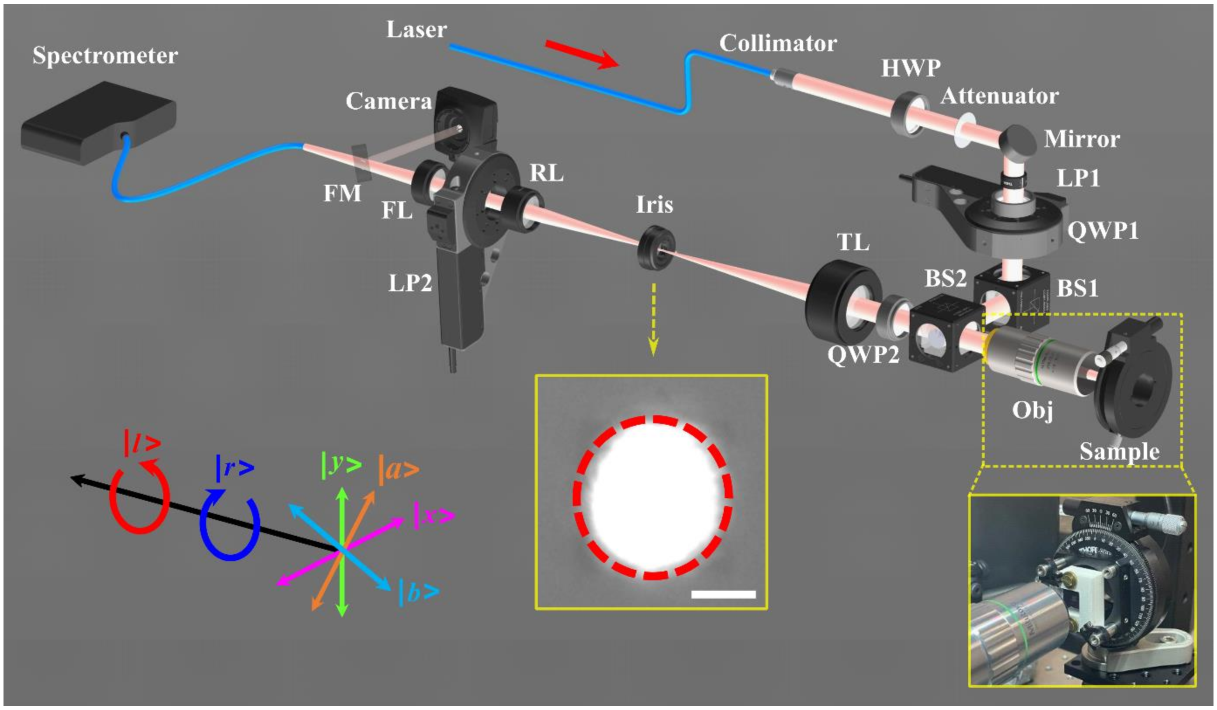

Fig. S4. Experimental setup for characterizing non-Hermitian MEMS metasurfaces. Laser: fiber-coupled supercontinuum laser (SuperK Extreme, NKT Photonics); Collimator: fiber collimator (TC06APC-780, Thorlabs); HWP: half-wave plate (AHWP10M-980, Thorlabs); Attenuator: absorptive neutral density filter (NE01B, Thorlabs); Mirror: protected sliver mirror (PF10-03-P01, Thorlabs); LP1 and LP2: linear polarizer (LPNIR050-MP2, Thorlabs); QWP1 and QWP2: quarter-wave plate (AQWP10M-980, Thorlabs); BS1 and BS2: beam splitters (CCM1-BS014/M, Thorlabs); Obj: long working distance objective (M Plan Apo 20×/0.42NA, Mitutoyo); TL: tube lens (TTL200-S8, f = 200 mm, Thorlabs); Iris: spatial filter (SM1D12SZ, Thorlabs); RL: relay lens (AC254-200-B-ML, f = 200 mm, Thorlabs); Camera: CMOS camera (DCC1545M, Thorlabs); FM: flip mirror (PF10-03-P01, Thorlabs); FL: flip lens (AC254-100-B-ML, f = 100 mm, Thorlabs); Spectrometer (QE Pro, Ocean Optics).

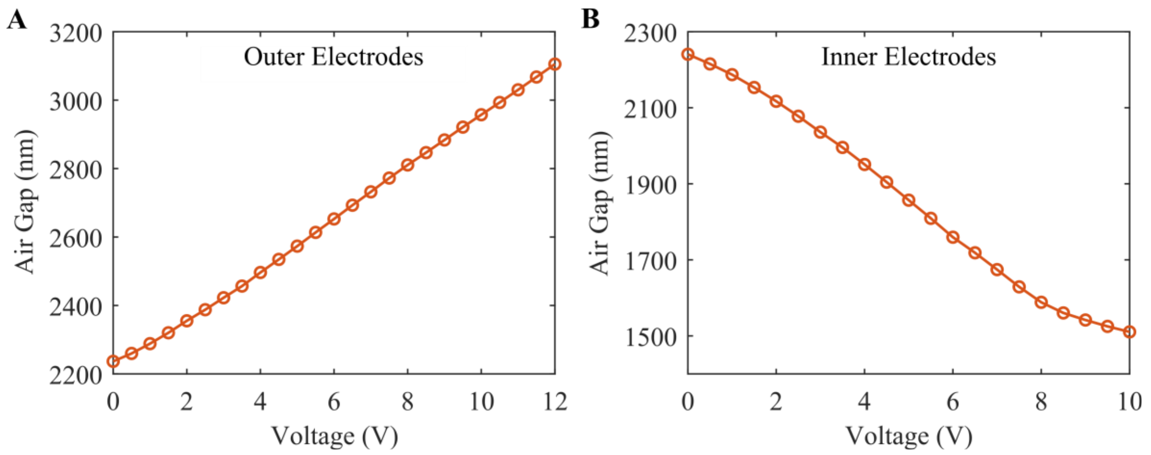

Fig. S5. Estimated air gap as a function of the voltage applied to the outer (A) and inner (B) electrodes. The outer electrodes enable quasi-linear movement for all voltages ranging from 0 V to 12 V, while the inner electrodes exhibit a nonlinear response for large voltages above 8 V.

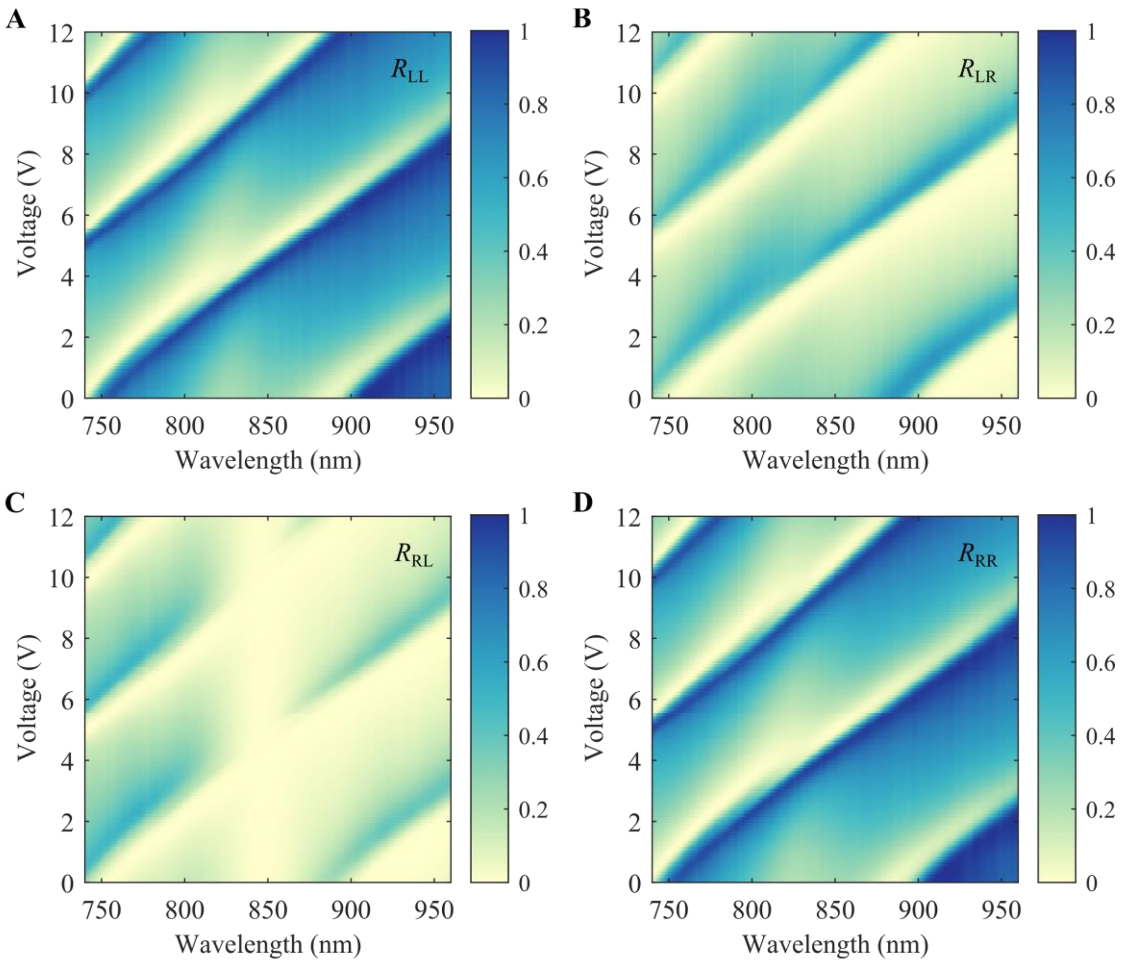

Fig. S6. Measured polarization-resolved reflectance R_{LL} (A), R_{LR} (B), R_{RL} (C), and R_{RR} (D) under LCP and RCP incidence when four outer electrodes are actuated from 0 V to 12 V in a step of 0.1 V.

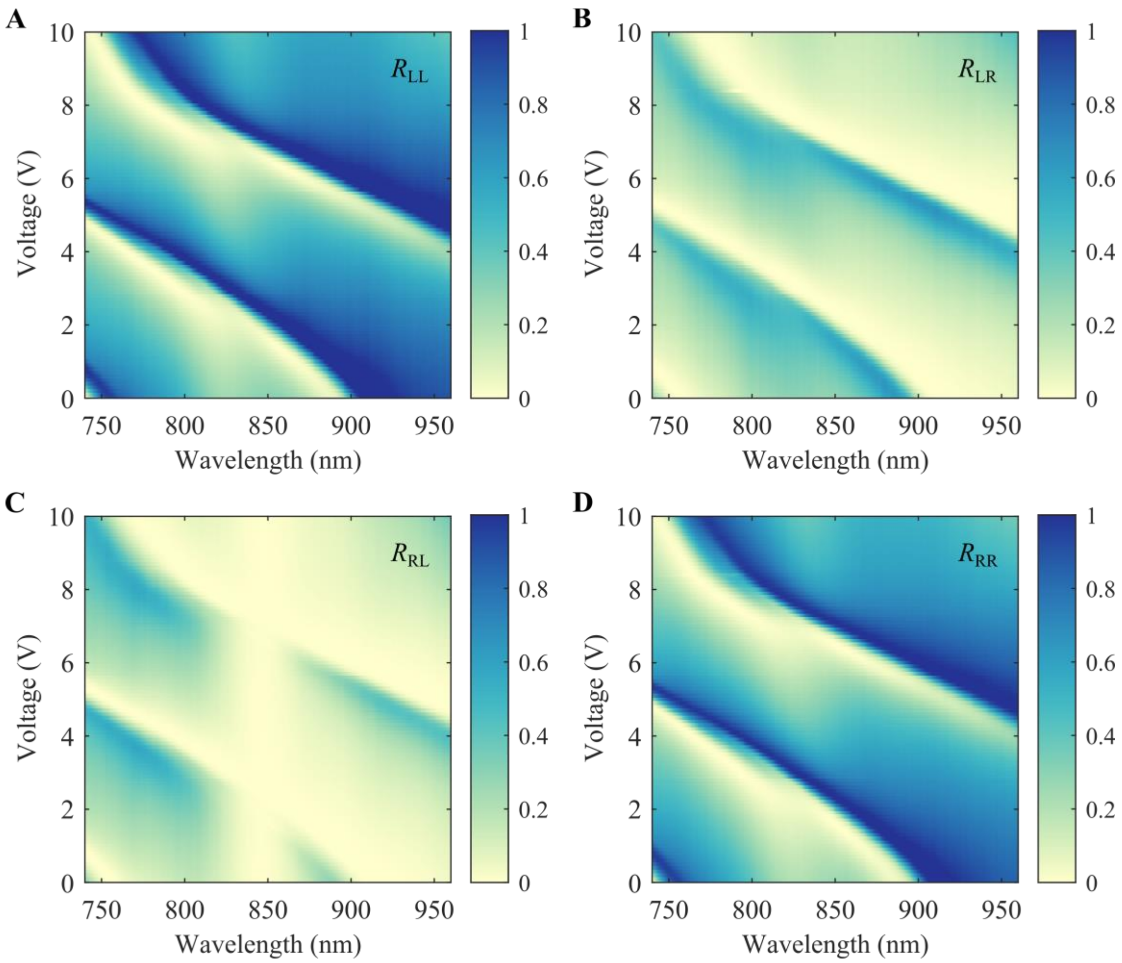

Fig. S7. Measured polarization-resolved reflectance R_{LL} (A), R_{LR} (B), R_{RL} (C), and R_{RR} (D) under LCP and RCP incidence when four inner electrodes are actuated from 0 V to 10 V in a step of 0.1 V.

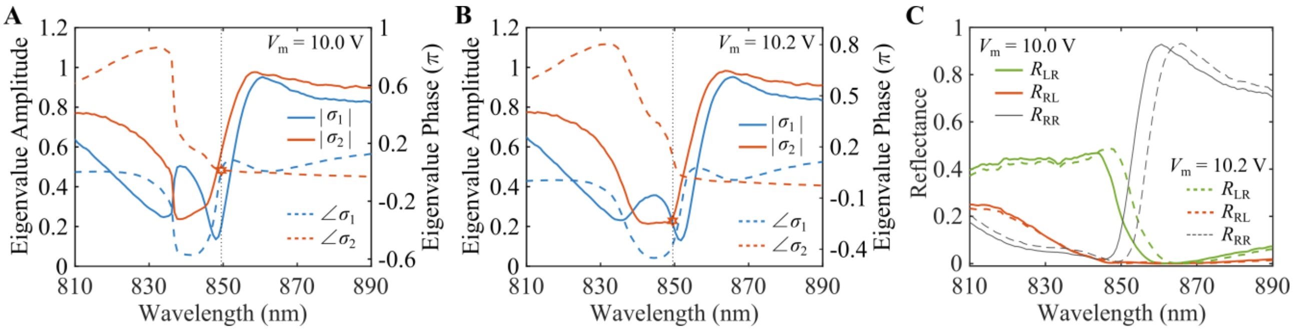

Fig. S8. Experimental observation of another chiral EP at λ = 849.505 nm when four outer electrodes are actuated. Measured eigenvalues (A and B) and reflectance (C) as a function of wavelength at two different voltages of V_m = 10.0 V and 10.2 V when driving four outer electrodes. Anti-crossing of eigenvalue amplitudes and crossing of eigenvalue phases are observed for V_m = 10.0 V, while crossing of eigenvalue amplitudes and anti-crossing of eigenvalue phases are observed for V_m = 10.2 V, revealing a chiral EP singularity at λ = 849.505 nm for V_m between 10.0 V and 10.2 V. The estimated air gaps t_a at the voltages of V_m = 10.0 V and 10.2 V are ~ 2957.2 nm and ~ 2975.1 nm, respectively.

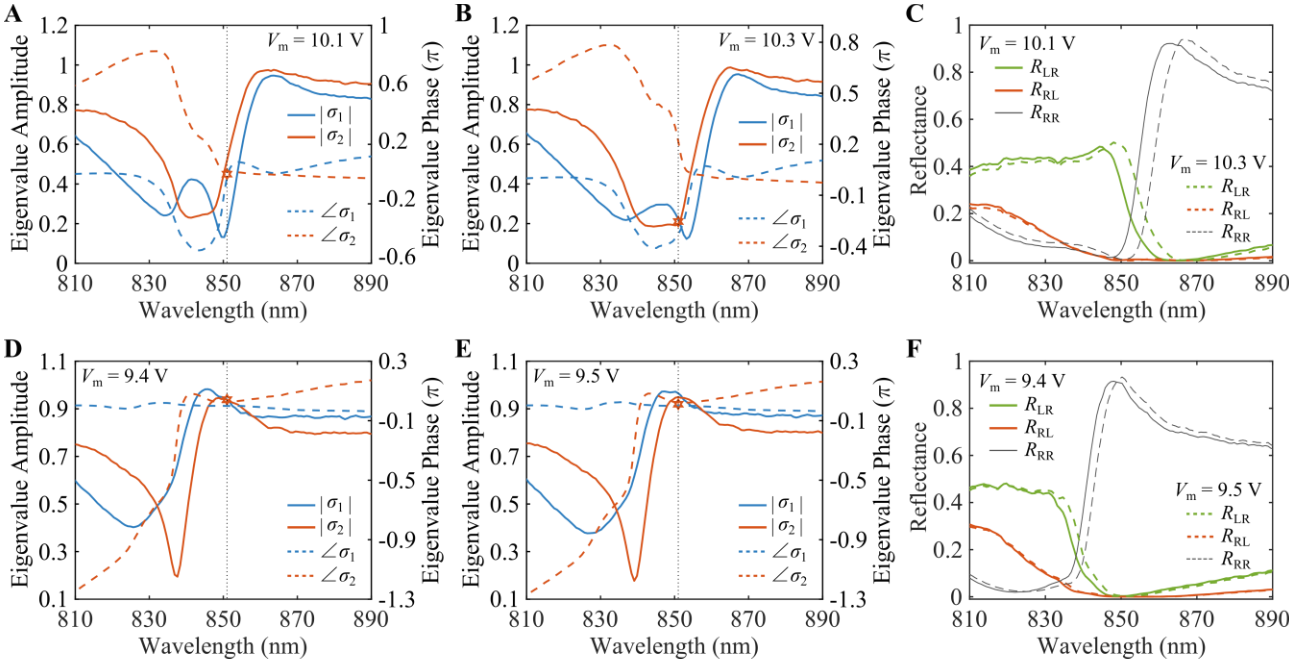

Fig. S9. Experimental observation of the dynamic topological transition from a chiral EP to a DP at λ = 850.991 nm when four outer electrodes are actuated. (**A** to **C**) Measured eigenvalues (A and B) and reflectance (C) as a function of wavelength at two different voltages of V_m = 10.1 V and 10.3 V when driving four outer electrodes. Anti-crossing of eigenvalue amplitudes and crossing of eigenvalue phases are observed for V_m = 10.1 V, while crossing of eigenvalue amplitudes and anti-crossing of eigenvalue phases are observed for V_m = 10.3 V, revealing a chiral EP singularity at λ = 850.991 nm for V_m between 10.1 V and 10.3 V. (**D** to **F**) Measured eigenvalues (D and E) and reflectance (F) as a function of wavelength at two different voltages of V_m = 9.4 V and 9.5 V. Crossing of eigenvalue amplitudes is observed for V_m = 9.4 V, indicating a DP at λ = 850.991 nm for V_m of ~ 9.4 V. The estimated air gaps t_a at the voltages of V_m = 9.4 V, 9.5 V, 10.1 V, and 10.3 V are ~ 2913.4 nm, ~ 2920.3 nm, ~ 2966.0 nm, and ~ 2981.1 nm, respectively.

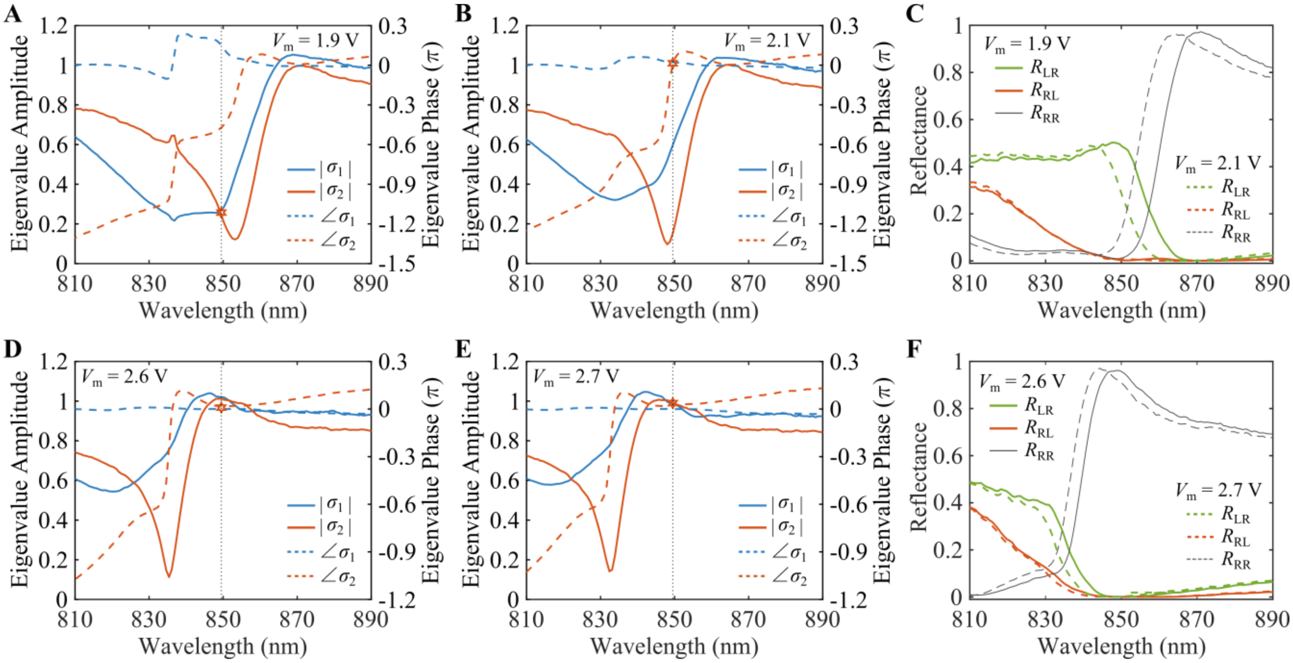

Fig. S10. Experimental observation of the dynamic topological transition from a chiral EP to a DP at λ = 849.505 nm when four inner electrodes are actuated. (**A** to **C**) Measured eigenvalues (A and B) and reflectance (C) as a function of wavelength at two different voltages of V_m = 1.9 V and 2.1 V. Crossing of eigenvalue amplitudes and anti-crossing of eigenvalue phases are observed for V_m = 1.9 V, while anti-crossing of eigenvalue amplitudes and crossing of eigenvalue phases are observed for V_m = 2.1 V, revealing a chiral EP singularity at λ = 849.505 nm for V_m between 1.9 V and 2.1 V. (**D** to **F**) Measured eigenvalues (D and E) and reflectance (F) as a function of wavelength at two different voltages of V_m = 2.6 V and 2.7 V. Crossing of eigenvalue amplitudes is observed for V_m = 2.7 V, indicating a DP at λ = 849.505 nm for V_m of ~ 2.7 V. The estimated air gaps t_a at the voltages of V_m = 1.9 V, 2.1 V, 2.6 V, and 2.7 V are ~ 2124.9 nm, ~ 2109.6 nm, ~ 2071.5 nm, and ~ 2063.9 nm, respectively.

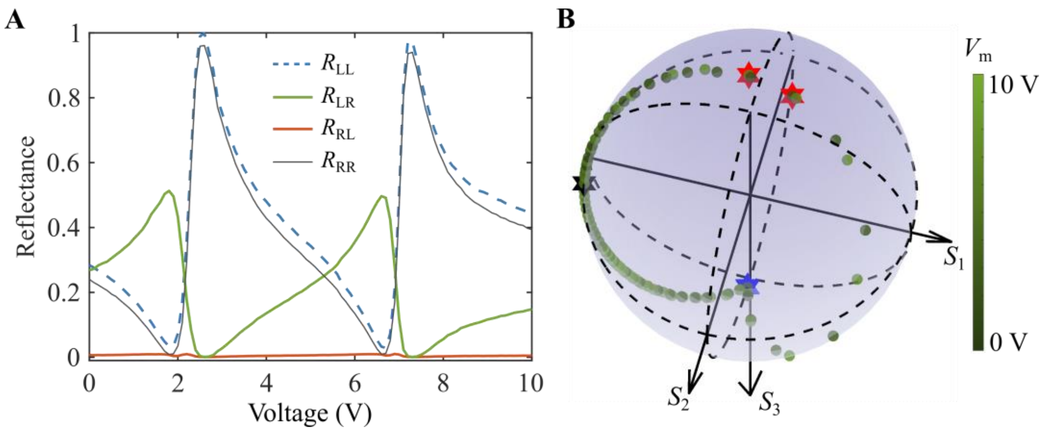

Fig. S11. Voltage-controlled polarization evolution at λ = 849.505 nm when four inner electrodes are actuated. (**A**) Measured reflectance as a function of the applied voltage. (**B**) Voltage-controlled polarization trajectory mapped on the Poincaré sphere for RCP incidence.

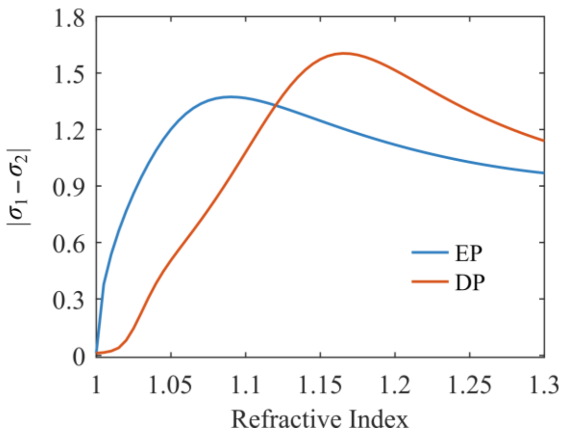

Fig. S12. Sensing with the non-Hermitian MEMS metasurface. The simulated absolute value of the difference of two eigenvalues as a function of the refractive index of the chemical that fills the gap between the chiral gold array and MEMS mirror. The EP mode shows higher sensitivity for ultra-small refractive index changes, while the DP mode is more sensitive to large changes. We could selectively drive the non-Hermitian MEMS sensor to work in the EP or DP mode to detect chemicals at any concentration with high sensitivity. The wavelength, air gap, and dimensions of the chiral meta-atom are the same as those in the main text.

Movie S1. Polarization-resolved optical images for dynamic topological EP-DP transition at λ = 849.505 nm when four outer electrodes are actuated with alternating voltages at 1 Hz.

Movie S2. Polarization-resolved Fourier images for dynamic topological EP-DP transition at λ = 849.505 nm when four outer electrodes are actuated with alternating voltages at 1 Hz.
